# Meat Quality Differences Between Ganan Tibetan Sheep and Tianzhu Tibetan Sheep Using Metabolomics and Rumen Microbiota Analyses

**DOI:** 10.3390/microorganisms14030575

**Published:** 2026-03-03

**Authors:** Yayuan Yang, Xindong Luo, Di Lu, Pengcheng Du, Sanye Jier, Xiaohu Wu, Yanan Lv, Pengcheng Dong, Xuezhi Ding

**Affiliations:** 1 Key Laboratory of Veterinary Pharmaceutical Development, Ministry of Agricultural and Rural Affairs, Lanzhou Institute of Husbandry and Pharmaceutical Sciences, Chinese Academy of Agricultural Sciences, Lanzhou 730050, China; 2 The First Clinical Medical College, Gansu University of Chinese Medicine, Lanzhou 730000, China

**Keywords:** Tibetan sheep, meat quality, rumen microbiota, lipid metabolomics

## Abstract

The objective of this study was to investigate the relationships between ruminal microbial communities and carcass traits associated with adipose accumulation in two Tibetan sheep breeds—Gannan and Tianzhu. A total of twenty Tibetan sheep (ten from each breed) were slaughtered, and samples of ruminal contents along with carcass trait data were collected for analysis. Ruminal microbial DNA was analyzed by 16S rRNA gene sequencing, and correlations between microbial composition and carcass traits were examined using correlation analysis and one-way ANOVA. The results showed that marbling score (*p* = 0.001) and longissimus lipid content (*p* = 0.007) were positively correlated with the Chao1 richness index, indicating that individuals with higher intramuscular fat content had greater ruminal microbial species richness. At the phylum level, Rikenellaceae RC9 gut group, Ruminococcaceae NK4A214 group were negatively correlated (*p* ≤ 0.05) with the above fat traits, whereas the abundance of the bacterial family Ruminococcus 1 was positively correlated with marbling score (*p* = 0.002). Stratified analysis by marbling grade further revealed associations with microbial richness (*p* ≤ 0.063), diversity (*p* = 0.044), and Ruminococcus 1 abundance (*p* < 0.001). However, microbial metabolic pathway prediction showed no significant differences (*p* ≥ 0.05) between the high- and low-marbling groups. In addition, several microbial taxa were positively correlated (*p* ≤ 0.05) with rib fat thickness and yield grade. In summary, ruminal microbial composition was closely associated with variations in carcass fat traits. Notably, most of the bacterial taxa associated with intramuscular and subcutaneous fat deposition did not overlap, suggesting that microbial metabolites may regulate fat deposition by influencing distinct adipogenic pathways in the host.

## 1. Introduction

Tibetan sheep are a primitive breed native to the Qinghai–Tibet Plateau, typically residing at elevations between 3000 and 5000 m. Tibetan sheep are a unique local breed formed through long-term adaptation to the cold and high-altitude environments [1]. For instance, the Oula type of Tibetan sheep inherently exhibits low-fat1and low-cholesterol characteristics. Tibetan sheep are raised in a semi-wild manner on the plateau, which involves high levels of physical activity and energy expenditure [2]. They forage on natural, high-quality pastures and various medicinal plants, a diet that naturally contributes to the development of low-fat meat [3]. The primary differences in meat quality between Tianzhu Tibetan sheep and Gannan Tibetan sheep originate from their distinct genetic adaptations to altitude-driven environmental conditions, which manifest as differential emphasis on meat tenderness and fat metabolism [4,5]. Investigating the relationship between rumen function and meat quality aims to unravel the mechanisms by which these sheep convert coarse forage into high-quality meat, a process rooted in digestive physiology, ultimately informing strategies for breed selection and precision feeding. Currently, there is a scarcity of direct comparative studies evaluating the meat quality of “Tianzhu Tibetan sheep” and “Gannan Tibetan sheep” as two well-defined populations. Research indicates that there are differences in fat deposition and fatty acid composition among different skeletal muscles of Tibetan sheep [6]. For example, the n-6/n-3 polyunsaturated fatty acid ratio in the *longissimus lumborum* is more ideal, closer to the optimal dietary ratio. The diversity of the rumen microbiota and the substantial influence of its ecological niches, functional groups, and metabolic networks on fermentation products and host outcomes make a systematic evaluation of this community essential for understanding how compositional shifts affect fermentation outputs and host traits such as milk, meat, and fiber production [1].

Emerging evidence suggests that the ruminal microbiome is closely linked to ruminant production traits, including milk fatty acids (FA) composition [7], feed efficiency [8,9,10,11,12], and methane emissions [13,14]. The gut microbiota and its metabolites significantly regulate host lipid metabolism and influence intramuscular fat (IMF) deposition [15]. Traditionally, gut microbes have been shown to interact with diet to modulate lipid metabolism [16] and composition [17]. More recent studies have revealed that functional microbial metabolites—such as short-chain fatty acids (SCFAs), bile acids (BAs), lipopolysaccharides (LPS), trimethylamines (TMA), and tryptophan derivatives—play key roles in host fat metabolism. SCFAs, in particular, modulate lipid metabolism through several mechanisms: (1) serving as auxiliary energy sources, (2) acting as signaling molecules via G-protein-coupled Receptor 43 (GPR43) and G protein coupled Receptor 41 (GPR41) receptors in white adipose tissue (WAT) and L-cells, and (3) stimulating hepatic and adipose Free Fatty Acid Receptor γ (PPARγ) pathways through butyrate and propionate activity [18]. Given these findings, the relative abundance (RA) of specific microbes is closely associated with phenotypic traits, underscoring the influence of the ruminal microbiota on animal performance. Recent advances in high-throughput and precise technologies have deepened our understanding of host–microbiota interactions. However, despite established correlations, the specific relationship between the rumen microbiota and lamb IMF remains underexplored, even though improvements in production efficiency have significantly benefited lamb producers [19]. It is widely recognized that lamb carcass value is largely determined by consumer preferences, which ultimately dictate economic returns; therefore, improvements in carcass quality and yield benefit producers and consumers alike.

Based on previous research, we hypothesized that differences exist in the composition of rumen microbiota and muscle metabolites between Tibetan sheep breeds, and that interactions may occur between these two factors [20]. This study aimed to preliminarily investigate the relationships among rumen microbial populations, all metabolites in the *longissimus lumborum* muscle, and yield traits in Gannan and Tianzhu sheep. We hypothesized that the two breeds, which differ in carcass merit and the quantified content of amino and fatty acids, would harbor distinct ruminal microbiota. These compositional differences were further hypothesized to alter the expression of key microbial metabolic pathways.

## 2. Materials and Methods

### 2.1. Experimental Animals

A total of twenty healthy, weaned lambs (100 days of age) with an average body weight of 18.00 ± 3.00 kg were selected from a uniform pasture in Gannan Tibetan Autonomous Prefecture. These lambs were randomly assigned to one of two dietary treatments: the TZ group (Tibetan Tianzhu sheep, *n* = 10) or the GN group (Tibetan Gannan sheep, *n* = 10). All experimental animals were co-housed in a single sheepfold equipped with a dry, well-ventilated, and sunlit exercise area under shelter. Subsequent to a 7-day pretrial adaptation phase, a 90-day fattening period was initiated, during which feed was provided twice per day at 08:30 and 16:30. The dietary regimen adhered to the “Feeding standard of meat-producing sheep and goats” (NY/T 816-2021) China Agriculture Press: Beijing, China, 2021, comprising a concentrate-to-forage ratio of 7:3; the forage component was an equivalent mixture of oat hay and corn silage.

In accordance with animal welfare guidelines [21], a 12 h fasting period was implemented prior to slaughter. Humane slaughter was conducted following standard protocols at a commercial abattoir. The *longissimus lumborum* muscle (from the 9th to 11th rib region) and rumen fluid samples were collected under strict supervision. All samples were snap-frozen and stored at −80 °C until analysis. For data collection, a consistent number of ten biological replicates per group were used for all assays, including meat quality evaluation, 16S rDNA sequencing, and metabolomics analysis.

### 2.2. Meat Quality Assessment

Meat quality parameters were assessed 24 h post-slaughter using standard protocols. Loin pH was measured at designated time points using a Testo^®^ 230 m (Testo GmbH & Co., Lenzkirch, Germany). The pH meter was calibrated according to established procedures [22], using standard buffer solutions (pH 4.0 and 7.0) stored at 20 °C (Mallinckrodt Chemicals, Phillipsburg, NJ, USA).

Meat color was evaluated following the method using a Minolta colorimeter (Model CR 300, Minolta Camera Co., Ltd., Osaka, Japan). Color measurements were based on the CIE L*, a*, and b* system, corresponding to lightness (L*), redness (a*), and yellowness (b*), using a 0°/45° geometry. Samples were removed from vacuum packaging 30 min prior to measurement, and surfaces were exposed to air to promote myoglobin oxygenation [23]. Meat color was recorded at three time points, and the average value was used for analysis.

Cooking loss was determined by calculating the proportion of water lost relative to the initial sample weight. Each sample was vacuum-sealed and cooked in batches of the same group for 30 min in a thermostatic water bath at 85 °C [24].

Thaw loss and drip loss were evaluated using the same principle as cooking loss. For drip loss, meat samples were suspended in a refrigerator at 4 °C for 24 h. To assess thaw loss, samples (average weight: 25 g) were thawed in a refrigerator at 4 °C for 12 h [25].

### 2.3. Fatty Acid (FA) Composition Analysis

For fatty acid (FA) analysis, 50 mg of meat sample was combined with 1 mL of chloroform-methanol solution in a 2 mL glass centrifuge tube and sonicated for 30 min [26]. The supernatant was subjected to methyl esterification using 2 mL of 1% sulfuric acid in methanol at aqueous conditions for 30 min. Fatty acid methyl esters were extracted with 1 mL of n-hexane, washed with 5 mL of pure water, and supplemented with 25 µL of methyl salicylate as an internal standard. A 500 µL aliquot of the supernatant was analyzed by GC-MS with a 10:1 split ratio.

Separation was achieved using an Agilent GC system equipped with a DB-WAX capillary column (Agilent Technologies, Wilmington, DE, USA) (30 m × 0.25 mm × 0.25 µm). The temperature program was as follows: hold at 40 °C for 5 min, ramp to 220 °C at 10 °C/min, and maintain at 220 °C for 5 min. Helium carrier gas was used at a constant flow rate of 1.0 mL/min. The inlet, ion source, and transfer line temperatures were set at 280 °C, 230 °C, and 250 °C, respectively. Mass spectrometry was conducted in single ion monitoring (SIM) mode with electron impact ionization (70 eV). QC samples were processed identically to experimental samples.

Metabolite qualification and quantification were performed using MultiQuant (version 3.0.3) and MSD ChemStation software (version 01.10) (Agilent Technologies, Santa Clara, CA, USA) for FAs, respectively [10,11]. These platforms enabled automated retention time correction, peak detection, and chromatographic alignment.

### 2.4. Short-Chain FAs Evaluation

For short-chain fatty acid (SCFA) analysis, rumen liquor (1 mL) was acidified with 1 mL of 0.5 M sulfuric acid. After centrifugation, 1 mL of the supernatant was extracted with 2 mL of diethyl ether via vigorous vortexing (2 min), followed by centrifugation (1484.7× *g*, 10 min) and incubation (4 °C, 30 min). The ether layer was stored at −20 °C prior to chromatographic analysis. Analysis was conducted using gas chromatography-mass spectrometry (GC-MS) on a Thermo 1300 system coupled with an ISQ ion trap MS. Samples (1 μL) were injected into a TR-WAXMS capillary column (30 m × 0.25 mm i.d. × 0.25 μm; Thermo Fisher Scientific, Waltham, MA, USA) with a split ratio of 10:1. High-purity helium served as the carrier gas at a constant flow rate of 1.2 mL/min. The GC oven temperature was programmed from 100 °C (hold 0.5 min) to 180 °C at 8 °C/min (hold 1 min), then to 200 °C at 20 °C/min, with a final hold at 200 °C for 5 min. Both the injector and ion source were maintained at 250 °C. Mass detection covered a range of *m*/*z* 40–450 in full-scan mode. SCFAs were quantified based on their characteristic retention times [26].

### 2.5. Meat Metabolomics Profiling

Meat samples were first chilled in a 2:1:1 (*v*/*v*) mixture of methanol: acetonitrile: water, then vortexed for 60 s and sonicated for 30 min. Following centrifugation at 14,000× *g* for 20 min, the samples were incubated at −20 °C for 10 min. The resulting supernatant was removed, and the pellet was resuspended in 100 µL of acetonitrile:water (1:1, *v*/*v*) for UHPLC-QTOF-MS analysis using a 1290 Infinity system (Agilent Technologies, Santa Clara, CA, USA). For chromatographic separation, 2 µL of each sample was injected using an autosampler (kept at 4 °C), with a flow rate of 0.5 mL/min and a column temperature of 25 °C. Mobile phase A consisted of 25 mM ammonium acetate and ammonium in water, and mobile phase B was acetonitrile. The gradient elution program lasted 2.9 min: 95% B for the first 0.5 min, linearly decreased to 65% from 0.5 to 7 min, further reduced to 40% over 1 min, then returned to 95% within 0.1 min. Randomized sample injections were performed to minimize signal drift, and quality control (QC) samples were included throughout the sequence to monitor system stability and reliability [27].

Mass spectrometry was conducted using an AB Triple TOF 6600 system. The following electrospray ionization (ESI) parameters were applied: Ion Source Gas 1 and Gas 2 at 60, source temperature at 600 °C, curtain gas (CUR) at 30, and ion spray voltage floating (ISVF) at 5500. The TOF-MS scan range was *m*/*z* 25–1000, and the product ion scan range was *m*/*z* 60–1000. Accumulation times were 0.20 s/spectrum for TOF-MS and 0.05 s/spectrum for product ion scans. Data-dependent acquisition (IDA) with enhanced sensitivity mode was used to acquire MS/MS spectra [28].

Data processing, including peak detection, retention time correction, and alignment, was performed using XCMS (version 3.7.1) (The Scripps Research Institute, La Jolla, CA, USA) [29]. Heat maps were generated using R software (version 4.5.2) (R Foundation for Statistical Computing, Vienna, Austria. URL https://www.R-project.org/), and multivariate statistical analyses were conducted using SIMCA (version 14.0), including principal component analysis (PCA), orthogonal partial least squares discriminant analysis (OPLS-DA), and partial least squares discriminant analysis (PLS-DA). Differential metabolites (DMs) were identified based on a variable importance in projection (VIP) score > 1 from the OPLS-DA model and a *p*-value < 0.05 from univariate statistical tests. Fold changes were calculated by comparing mean metabolite levels between the GN and TZ groups. Functional annotation and metabolic pathway enrichment were performed using the KEGG database [30].

### 2.6. Ruman Microbiome

Rumen microbiota analysis was performed following a previously published protocol [31]. Genomic DNA was extracted from 0.25 g of rumen content using the PowerSoil DNA Isolation Kit (MO BIO Laboratories, Carlsbad, CA, USA), according to the manufacturer’s instructions. The V3–V4 region of the bacterial 16S rRNA gene was amplified and purified, and sequencing was carried out using the Illumina HiSeq platform (Applied Protein Technology, Shanghai, China). All PCR reactions were carried out in 30 μL reactions with 15 μL of Phusion^®^High-Fidelity PCR Master Mix (New England Biolabs, Ipswich, MA, USA); 0.2μM of forward and reverse primers, and about 10 ng template DNA. Thermal cycling consisted of initial denaturation at 98 °C for 1 min, followed by 30 cycles of denaturation at 98 °C for 10 s, annealing at 50 °C for 30 s, and elongation at 72 °C for 60 s. Finally, 72 °C for 5 min. Raw sequence data were deposited in the BIGD Genome Sequence Archive under accession number CRA001467. Sequencing libraries were assessed with an Agilent 2100 Bioanalyzer (Agilent Technologies, Santa Clara, CA, USA), and qualified libraries were amplified on a cBot to generate clusters on a flow cell. Samples were indexed with unique barcodes and subjected to paired-end sequencing. Overlapping paired-end reads were merged using FLASH v1.2.7. Low-quality reads were removed according to QIIME (v1.7.0) quality control standards to obtain high-quality clean tags. Chimeric sequences were detected and removed using the UCHIME [MOTHUR v1.] algorithm by comparison with the Gold database. Bioinformatic analyses were conducted using QIIME 2 (version 2023.9). Sequences with ≥97% similarity were grouped into operational taxonomic units (OTUs). Alpha diversity, beta diversity, and principal coordinates analysis (PCoA) were calculated using unweighted UniFrac distances.

### 2.7. Statistical Analysis

Data distribution was assessed using the Kolmogorov–Smirnov test. Results were expressed as mean ± SEM. Differences between groups were evaluated using Student’s *t*-test or the Wilcoxon rank-sum test, with statistical significance defined as follows: *p* < 0.05, *p* < 0.01, and *p* < 0.005. A threshold of *p* < 0.05 was considered statistically significant.

## 3. Results

### 3.1. Edible Quality and Meat Nutritional Components

Our findings indicated that natural grazing in different regions influenced several meat quality parameters, including pH, shear force, drip loss, color, final pH, and moisture content (Table 1). Specifically, both initial (pH_0_) and 24 h postmortem pH (pH_24_) values were lower in the TZ group compared to the GN group. Meat color is a key sensory attribute used by consumers to assess freshness and quality. As shown in Table 1, the lightness (L*) value was higher in the TZ group, whereas redness (a*) and yellowness (b*) values were lower. However, these differences were not statistically significant (*p* > 0.05). The redness value is particularly indicative of meat color quality [20]. No significant difference was observed in cooking loss between the TZ and GN groups. Cooking loss generally correlates positively with intramuscular fat content, with grass-fed mutton typically exhibiting higher loss. However, tenderness remained consistent across grazing regions [21,22,23]. These findings suggest that the GN group may have experienced a slower pH decline in the *longissimus lumborum* (LL) muscle within 24 h postmortem, potentially enhancing water-holding capacity (WHC) and texture in the TZ group [22].

### 3.2. FA Compositions

Regional differences in fatty acid composition of the LL muscle in naturally grazed Tibetan sheep are presented in Table 2. The distribution and content of fatty acids are key indicators of meat quality and have significant implications for human health. Fatty acid profiling of the *longissimus lumborum* muscle identified 17 compounds, comprising 5 saturated fatty acids (SFAs), 3 monounsaturated fatty acids (MUFAs), and 9 polyunsaturated fatty acids (PUFAs). The principal fatty acids were palmitic acid (C16:0), stearic acid (C18:0), and oleic acid (C18:1), which together accounted for 80–86% of the total fatty acid content. Oleic acid was the most abundant, followed by palmitic acid. Compared with the TZ group, the GN group showed significantly higher SFA (48.01% vs. 46.19%) and MUFA (45.51% vs. 42.11%) levels (*p* < 0.01), but a lower PUFA content (7.17% vs. 10.92%; *p* < 0.05). The major SFAs identified were C16:0 and C18:0. Although the GN group exhibited slightly lower levels of C14:0, C16:0, and C18:0 relative to the TZ group, these differences were not statistically significant. The relatively reduced SFA content in the TZ group may be linked to differences in gastrointestinal microbial communities, which could limit SFA deposition and thereby improve the nutritional quality of the meat. Oleic acid was the predominant MUFA, comprising up to 40% of total fatty acids, followed by cardamoic acid and palmitoleic acid. No significant differences were observed between groups in the MUFA profile (*p* > 0.05).

The TZ group exhibited a significantly higher overall PUFA content compared to the GN group. Specifically, the concentrations of linoleic acid, eicosapentaenoic acid (EPA), and docosahexaenoic acid (DHA) were all significantly elevated in the TZ group (*p* < 0.05). Notably, the TZ group also showed a 0.06% increase in conjugated linoleic acid (CLA), a fatty acid known for its role in lipid metabolism and high biological activity. In particular, the n-3 PUFA content was significantly higher in the TZ group, while n-6 PUFA levels were lower, leading to a more favorable n-6/n-3 ratio—a recognized indicator of nutritional quality. The lower n-6/n-3 ratio in the TZ group thus indicates a higher nutritional value of the *longissimus lumborum* muscle compared to the GN group.

### 3.3. Short-Chain FAs

Natural grazing in different regions significantly influenced the concentrations of gut microbiota-derived metabolites. As shown in Table 3, acetic acid, butyric acid, and valeric acid levels were significantly higher in the TZ group than in the GN group (*p* < 0.05), while propionic acid and isovaleric acid levels were significantly higher in the GN group (*p* < 0.05). No significant difference was observed in isobutyric acid levels between the two groups (*p* > 0.05).

### 3.4. Muscular Metabolomics Analysis

**Figure 1:**
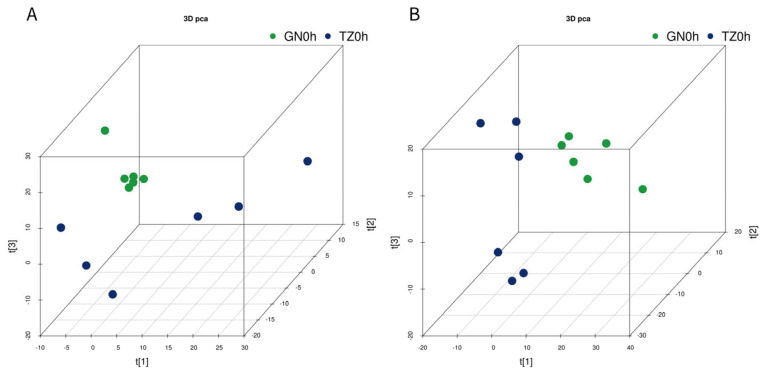
3D PCA score of the overall samples in the positive (**A**) and negative (**B**) ion detection mode. The GN group is marked as green and the TZ samples are indicated with blue (*n* = 6 per group).

**Figure 2:**
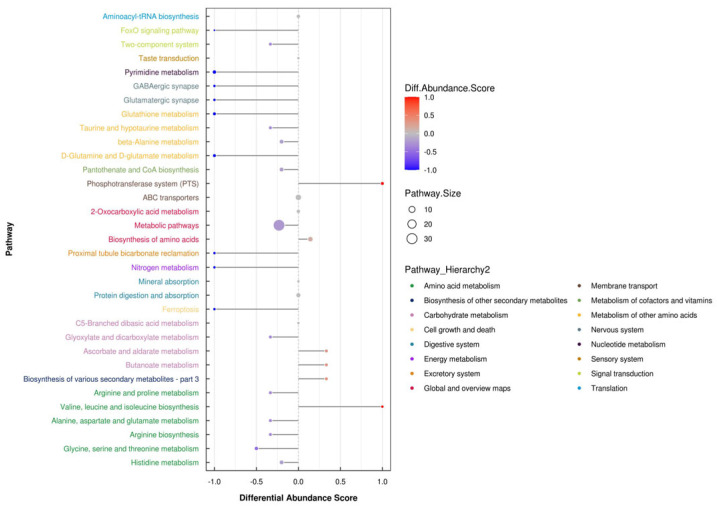
The overlap of differential metabolites linked to KEGG metabolic pathways in the *longissimus lumborum* of Tibetan sheep for both comparisons.

**Figure 3:**
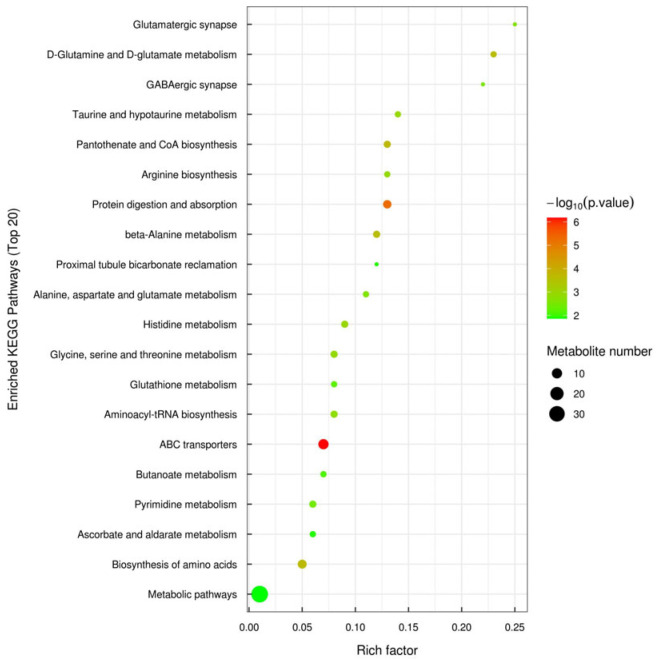
KEGG pathway enrichment analysis of differential metabolites in *longissimus lumborum* muscle between GN and TZ Tibetan sheep (*n* = 6 per group).

Three-dimensional PCA score plots of the two groups in both negative and positive ion modes are shown in Figure 1. These plots revealed clear separation between the GN and TZ groups, which was further confirmed by PLS-DA and OPLS-DA analyses (Supplementary Figures S1 and S2). The OPLS-DA results demonstrated distinct separation and clustering within and between the negative (R^2^X = 0.347, R^2^Y = 0.992, Q^2^ = 0.793) and positive (R^2^X = 0.524, R^2^Y = 0.981, Q^2^ = 0.861) ion modes. Additional analyses confirmed distinct separations between the GN and TZ groups in both negative (R^2^X = 0.347, R^2^Y = 0.992, Q^2^ = 0.908) and positive (R^2^X = 0.617, R^2^Y = 0.995, Q^2^ = 0.871) ionization modes. A total of 1724 differential metabolites (DMs) were identified. Among these, 46 key metabolites—including nucleotides, fatty acids, amino acids, organic acids, and carbohydrates—were mapped to KEGG pathways. KEGG pathway analysis indicated that differences between regions primarily affected nucleotide metabolism, transmembrane transport, lipid metabolism, amino acid metabolism, and carbohydrate metabolism (Figure 2). Metabolic networks such as proximal tubule bicarbonate reclamation, pyrimidine metabolism, phosphotransferase system (PTS), pantothenate and CoA biosynthesis, ether lipid metabolism, and carbohydrate digestion and absorption were significantly upregulated in the GN group. Conversely, pathways including 2-oxocarboxylic acid metabolism, ABC transporters, alanine, aspartate and glutamate metabolism, aminoacyl-tRNA biosynthesis, arginine biosynthesis, beta-alanine metabolism, biosynthesis of amino acids, biosynthesis of various secondary metabolites (part 3), and butanoate metabolism exhibited significant differences between groups (Figure 3). Furthermore, our data suggest that gut microbiota substantially influenced both the LL muscle PTS and AMPK signaling pathways in Tibetan sheep.

### 3.5. Characteristics of the Rumen Fermentation and Composition Analysis

**Figure 4:**
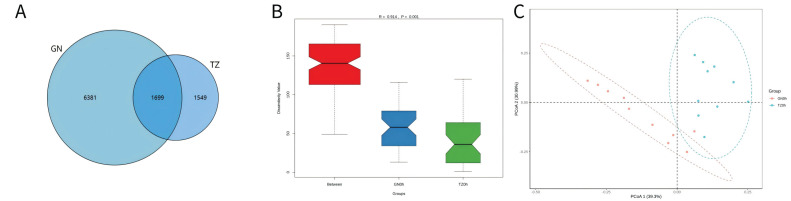

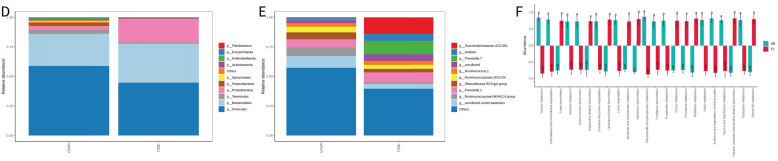
Analysis of Rumen Microbiota Structure and Function. (**A**) Venn diagram showing the number of unique and shared operational taxonomic units (OTUs) among the three experimental groups (*n* = 6 per group). (**B**) Analysis of Similarity (ANOSIM) test result, demonstrating significant microbial community differences between groups. (**C**) Principal Coordinates Analysis (PCoA) plot based on Bray–Curtis distances, illustrating the overall microbial community divergence between groups (the percentage of variance explained by each axis is indicated). (**D**,**E**) Relative abundance (%) of the dominant bacterial communities at the phylum (**D**) and genus (**E**) levels. Only the top 10 most abundant taxa are shown. (**F**) Box plot (*n* = 6 per group) depicting the predicted abundance of the most significantly differential rumen microbial function identified through metagenomic analysis.

An average of 41,320 sequence reads per sample (range: 27,856–46,419) was clustered into operational taxonomic units (OTUs). Our data showed that, compared to GN sheep, TZ sheep had significantly higher ACE (*p* = 0.001) and Chao (*p* = 0.001) indices. Consistent with these findings, significant differences were observed between the sheep in Shannon (*p* < 0.001) and Simpson (*p* = 0.004) indices, which showed strong agreement. Taxonomic analysis identified 19 phyla, with Bacteroidetes, Fibrobacteres, Firmicutes, Tenericutes, Synergistetes, Lentisphaerae, Spirochaetae, and Proteobacteria as the eight most abundant (Figure 4). Comparing GN and TZ sheep, Firmicutes RA was significantly lower in GN (*p* < 0.001), while Bacteroidetes RA was significantly higher in TZ (*p* < 0.001). Furthermore, TZ sheep had significantly higher RA of Butyrivibrio_2 (*p* < 0.001), Ruminococcaceae_UCG-002 (*p* < 0.001), Saccharofermentans (*p* = 0.003), and Succiniclasticum (*p* = 0.006) than GN sheep. PCoA using weighted UniFrac distances revealed marked differences in rumen microbiome composition between dietary groups. Substantial microbial differences existed between the two regions, as collectively evidenced by ANOSIM, which showed intergroup variation significantly exceeded intragroup variation (R = 0.7332, *p* = 0.001; Figure 4B).

### 3.6. SubsecCorrelation Between Muscle Metabolomics and Rumen Bacteria

**Figure 5:**
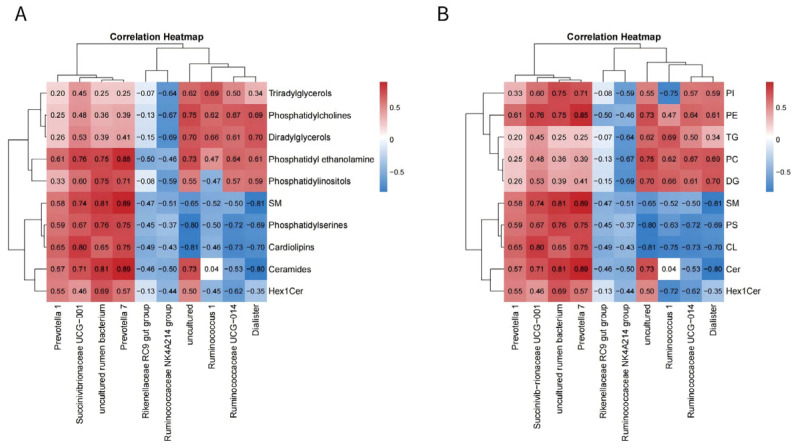
(**A**) The correlation heat map between meat quality parameters and muscle metabolomics analysis. (**B**) The correlation heat map between muscle metabolomics analysis and rumen bacteria. The color red and blue represent positive and negative correlations, respectively. G: glycerol; PE: phosphoethanolamine; GP(2): glycerophosphate(2); GPC: glycerophosphocholine (*n* = 6 per group).

This study investigated the correlation between rumen bacteria and muscle metabolism in naturally grazing Tibetan sheep from different regions by integrating metabolomic and phenotypic data from the LL. A significant association was observed between the two (Figure 5A). Correlation analyses further revealed that intramuscular fat content was positively correlated with a wide range of lipid classes. The levels of heterocycles, alcohols, and terpenoids, along with aldehydes and ketones, showed positive correlations with metabolites involved in purine and carbohydrate metabolism. Moreover, aldehydes were negatively correlated with the abundance of specific rumen bacteria, such as the *Rikenellaceae RC9* gut group and *Ruminococcaceae NK4A214 group*, while alcohol levels were positively correlated with key glycerophosphate compounds. Pathway analysis indicated that the regulation of purine and carbohydrate metabolism—including carbohydrate digestion and absorption, sucrose and starch metabolism, and galactose metabolism—significantly influenced the concentrations of these metabolites in the LL.

### 3.7. Correlation Between Muscle Fat Metabolomics and Rumen Bacteria

To further understand the regional adaptations, the analysis focused on the association between rumen microbiota and fat-related metabolites in the LL muscle of naturally grazing Tibetan sheep. Similar correlations were observed between muscle fat metabolites and rumen bacterial abundance (Figure 5B). Notably, *Prevotella 1*, *Succinivibrionaceae UCG-001*, and *Prevotella 7* levels positively correlated with fat metabolites. Conversely, *Rikenellaceae RC9* gut group, *Ruminococcaceae NK4A214 group, Ruminococcus 1, Ruminococcaceae UCG-014*, and Dialister negatively correlated with SM, PS, CL, Cer, and Hex1Cer levels. Additionally, Rikenellaceae RC9 gut group and Ruminococcaceae NK4A214 group negatively correlated with PI, PE, TG, PC, and DG. In contrast, *Ruminococcus 1, Ruminococcaceae UCG-014*, and Dialister positively correlated with PE, TG, PC, and DG levels.

## 4. Discussion

Through integrated analysis of the ruminal and hindgut microbiome and metabolome, we identified both microbe-dependent and host metabolism-dependent mechanisms underlying differences in growth and meat quality. A comprehensive network was constructed to illustrate the interactions among microbial composition, functional capacity, and metabolite profiles that distinguish these traits. Notable variations in bacterial ratios were observed between breeds. Combined 16S sequencing and metabolome sequencing technologies, this study investigated the environmental adaptability of the rumen microbiota in Tibetan sheep from the same region. This approach provides a valuable perspective for exploring sheep meat quality traits and offers new insights into the adaptive evolution of ruminants. Tibetan sheep are a unique local breed formed through long-term adaptation to cold and high-altitude environments. For example, the Oula type of Tibetan sheep is inherently characterized by low fat and low cholesterol levels. Tibetan sheep are semi-wild grazed on the snowy plateau, where high levels of physical activity lead to substantial energy expenditure. They forage on natural, high-quality pastures and various medicinal plants, a diet that naturally contributes to the formation of low-fat meat. Studies have shown that there are differences in fat deposition and fatty acid composition among different skeletal muscles in Tibetan sheep. For instance, the n-6/n-3 polyunsaturated fatty acid ratio in the *longissimus lumborum* is more ideal, closer to the optimal dietary ratio [3,16]. The fatty acid profile of lamb plays a critical role in determining its quality and economic value, as it directly influences the development of the meat’s characteristic gamey flavor. In this study, the primary fatty acids identified in the muscle of Gannan and Tianzhu lamb were oleic acid (C18:1 9c) and palmitic acid (C16:0) [18,19,20,21]. This composition aligns with that observed in the muscle of cage-fed Longling Yellow Goats, suggesting that breed is a major factor governing muscle fatty acid composition. Among the polyunsaturated fatty acids (PUFAs), linoleic acid (C18:2 cis) and alpha-linolenic acid (C18:3 n-3) are both essential fatty acids for humans. Therefore, a higher content of these PUFAs in muscle generally corresponds to greater nutritional value of the meat. In the present study, no significant difference was observed in the levels of linoleic acid and alpha-linolenic acid between the two feeding groups, indicating comparable nutritional value in terms of essential fatty acid supply. However, previous research has shown that n-3 PUFAs possess anti-cancer properties by inhibiting tumor cell growth, whereas n-6 PUFAs may promote tumor development. In this investigation, the grazing group exhibited higher levels of n-3 PUFAs and lower levels of n-6 PUFAs compared to the housed feeding group. These findings imply that grazing may enhance the anti-tumor potential of lamb and improve its overall nutritional profile. In the hindgut ecosystem, the *Firmicutes*-to-Bacteroidetes ratio was significantly higher in Tianzhu sheep compared to Gannan sheep, a proportion previously correlated with adiposity [22]. Concurrently, reduced abundance of Rikenellaceae members in Tan sheep was associated with enhanced fat deposition [22]. Experimental colonization with *Lachnospiraceae* demonstrated its capacity to promote visceral organ (including hepatic) hypertrophy while reducing plasma insulin concentrations [29]. These microbial variations collectively contribute to phenotypic differentiation, consistent with established research. *Saccharofermentans*, organisms associated with recalcitrant high-lignin dietary components [19], demonstrate metabolic capability in converting succinate to acetate and propionate [23]. While *Succinivibrionaceae*—identified as the dominant ruminal bacteria in Dorper sheep—are recognized succinate producers [24], no significant succinate accumulation was detected. This apparent discrepancy may reflect the multifunctional metabolic capacity of *Succinivibrionaceae*, which extends beyond succinate generation to include degradation of starch, hemicellulose, and xylan [25,26]. *Lactobacillus*, recognized as beneficial microbiota, contributes to homeostasis maintenance and energy metabolism through lactate production [25,26,27]. Similarly, Phascolarctobacterium represents another beneficial genus capable of generating short-chain fatty acids [14]. These microbial metabolites provide valuable substrates for lipogenesis [10]. The dynamic abundance patterns of *Lactobacillus* and *Phascolarctobacterium* indicate remarkable adaptive capacity in Tan sheep, though their specific beneficial roles in ovine physiology warrant further investigation.

At the phylum level, the rumen microbial communities of Tibetan sheep from the two regions showed significant differences, which became more pronounced at the genus and species levels. *Bacteroidetes* and *Firmicutes* were dominant in both regions. Several studies [30,31] have shown that *Bacteroidetes* and *Firmicutes* drive the rumen microenvironment through different microbial diversity and community compositions. The dominant bacterial genera in the rumen included *Prevotella*, *Selenomonas*, *Clostridium*, and *Bacteroides*. At the species level, the dominant bacterium in the TZ group was *Selenomonas_bovis* (4.35%), while in the GN group, it was *Prevotella_ruminicola* (2.94%), indicating clear differences between the two groups at the species level. Chai’s study [26] showed that Prevotella_ruminicola in the goat rumen was strongly correlated with cellulose degradation after feeding.

Carbohydrate-Active Enzymes (CAZymes) are essential for microbial communities to thrive in carbohydrate-rich environments such as animal guts, agricultural soils, forest soils, and marine sediments [31]. CAZymes are a class of enzymes that break down glycosidic bonds between polysaccharides. The complexity of polysaccharide structures determines the diversity of CAZymes required for degradation. Currently, CAZymes can be divided into six categories: glycoside hydrolases (GHs), glycosyltransferases (GTs), polysaccharide lyases (PLs), carbohydrate esterases (CEs), carbohydrate-binding modules (CBMs), and auxiliary redox reductases [31].

Ruminal energy harvesting and hindgut lipid metabolism represent distinguishing characteristics that vary considerably across sheep breeds. Fumarate supplementation in Tan sheep enhances propionate production in the rumen, thereby promoting gluconeogenesis and improving feed utilization efficiency [16]. In contrast, Dorper sheep exhibit abundant glycogen amino acids and their derivatives, indicating a greater reliance on amino acids as energy substrates [27]. This evidence suggests divergent ruminal fermentation patterns among different breeds. The hindgut metabolic network further reveals variations in porphyrin and iron metabolism. Iron participates in lipid metabolism and facilitates fatty acid uptake and lipid droplet formation in the liver through synergistic mechanisms [28,29,30]. The elevated levels of fatty acids including N-palmitoylserine and 2-oxohexadecanoic acid in Tianzhu sheep underscore breed-specific characteristics in lipid metabolism. Niacin and pyrimidines were identified as regulators of physiological functions, as demonstrated by microbial metabolite-phenotype networks. Correlation analyses revealed that niacin concentrations positively correlated with body weight but negatively associated with fat deposition. As precursors of NAD^+^ and NADP^+^, niacin plays crucial roles in both catabolic and anabolic redox reactions. Furthermore, niacin exerts antilipolytic effects through hydroxycarboxylic acid receptor 2-mediated mechanisms [31]. Dietary niacin supplementation has been shown to reduce plasma triglyceride and non-esterified fatty acid concentrations [32,33,34]. Distinct pyrimidine metabolism patterns emerged as notable features in the hindgut, with different nucleoside combinations influencing growth performance [35]. Our findings align with this observation. The inconsistencies reported across studies may be attributed to multiple factors including animal species, age, and feeding conditions—intrinsic, management, and environmental variables that collectively contribute to experimental variations.

This study showed that the functional annotations of the rumen microbiota in the two groups belonged to the same categories, indicating that the functions of the rumen microbiota were relatively stable, primarily concentrated in GHs, GTs, and CBMs. GHs can break glycosidic linkages through hydrolysis [36,37]. The results of this study demonstrated a significant increase in GH31 and a pronounced decline in GH24 and GH39 in the rumen carbohydrate enzymes of the two Tibetan sheep groups. These results indicate that the rumen of Tibetan sheep underwent significant changes in response to forage scarcity and carbohydrate degradation due to long-term drought stress, thereby adapting to selection pressure.

GTs catalyze the activation of sugar moieties, attaching them to specific receptor molecules to form glycosidic bonds, and play a crucial role in carbohydrate synthesis, as well as in the adaptability and pathogenicity of host microorganisms [38]. It is worth noting that in this study, GT13 was significantly upregulated in the rumen of Tibetan sheep from high-altitude cold regions, which may play an important role in the synthesis of carbohydrates such as glucose. This could serve as an enzymatic marker to distinguish whether an organism can survive in high-altitude cold regions. CEs primarily remove polysaccharide ester groups and participate in the degradation of carbohydrate side chains, promoting the cleavage of glycosidic bonds by GHs and PLs [39]. PLs can disrupt glycosidic bonds through a complex mechanism [40]. CBMs have no catalytic activity themselves but assist GHs, PLs, and other hydrolytic enzymes by anchoring CAZymes to the substrate surface, increasing contact time and surface area [41,42]. CBM57 (upregulated in arid regions) and CBM14 (downregulated in arid regions) play important roles in the response of sheep to drought stress. AAs can participate in the modification of lignin, thus breaking the barrier of plant biomass resistance to degradation and accelerating substrate hydrolysis [43].

Therefore, changes in rumen microbiota lead to alterations in microbial carbohydrate enzymes, thereby promoting feed degradation in Tibetan sheep under drought stress, particularly the hydrolysis and synthesis of carbohydrate substances. This accelerates energy metabolism and promotes fat deposition in Tibetan sheep under harsh environments, ultimately affecting their growth performance and production performance.

## 5. Conclusions

This study demonstrated that identifying metabolites in muscle and microbiota in the rumen provides valuable insight into meat quality changes in Tibetan sheep. The GN group exhibited superior water-holding capacity, tenderness, lower fat content, and higher amino acid levels compared to the TZ group. Correlation analysis between meat quality and metabolites showed that WBSF, color, and water-holding capacity were closely associated with metabolites such as D-glucose 6-phosphate, regulated through pathways related to glycolysis, including the phosphotransferase system. Additionally, cholesterol metabolism emerged as a potential pathway regulating fat deposition. Correlation analysis of gut microbes with meat quality and metabolites indicated that gut microbes influence muscle metabolites via sugar and amino acid metabolism in the rumen, thereby modulating meat quality. However, further research is needed to elucidate the specific molecular regulatory mechanisms. These findings provide a theoretical basis for future studies on meat quality regulation and support the development of high-quality Tibetan sheep in the region.

## Figures and Tables

**Table 1:** Effect of different in natural grazing at different regions on the edible quality and nutritional components of the *longissimus lumborum* of Tibetan sheep. Within a row, asterisks denote significant differences between the GN and TZ groups (* *p* < 0.05, ** *p* < 0.01) based on independent samples *t*-test.

Item	Group		SEM	*p*-Value
	TZ	GN		
pH	6.96	7.24 *	0.83	*p* < 0.05
Initial pH (0 h)	6.53	6.62 **	0.05	*p* < 0.01
Ultimate pH (24 h)	5.72	5.78	0.05	*p* > 0.05
L*	27.26	25.76	1.00	*p* > 0.05
a*	7.79	9.76	1.85	*p* > 0.05
b*	5.43	6.81	1.01	*p* > 0.05
Shear force (N)	10.23	9.07	1.09	*p* > 0.05
Cooking loss (%)	0.40	0.42	0.17	*p* > 0.05
Drip loss (%)	5.12	7.13 **	0.51	*p* < 0.01
Moisture	74.56	75.38	0.58	*p* > 0.05
Ash	1.01	1.05	0.96	*p* > 0.05

SEM: Stander error of mean; GN: Gannan; TZ: Tianzhu.

**Table 2:** Effects of natural grazing in different regions on the fatty acid composition in the *longissimus lumborum* muscle of Tibetan sheep (*n* = 6). Within a row, asterisks denote significant differences between the GN and TZ groups (* *p* < 0.05, ** *p* < 0.01) based on independent samples *t*-test.

Item	Group		SEM	*p*-Value
	TZ	GN		
C10:0	0.04	0.05	0.007	*p* > 0.05
C12:0	0.03	0.04	0.007	*p* > 0.05
C13:0	0.02	0.02	0	*p* > 0.05
C14:0 iso	0.24	0.16 *	0.056	*p* < 0.05
C14:0	1.32	1.65 **	0.233	*p* < 0.01
C14:1	0.11	0.06 **	0.035	*p* < 0.01
C15:0	0.14	0.01 **	0.092	*p* < 0.01
C15:0 anteiso	0.06	0.06	0	*p* > 0.05
C15:0	0.30	0.31	0.007	*p* > 0.05
16:0 iso	0.13	0.10	0.021	*p* > 0.05
C16:0	20.87	22.01	0.806	*p* > 0.05
C16:1 n9	0.18	0.36 **	0.127	*p* < 0.01
C17:0 iso	0.21	0.19	0.014	*p* > 0.05
C17:0 anteiso	2.33	2.55	0.155	*p* > 0.05
C17:0	2.25	2.38	0.091	*p* > 0.05
C17:1	1.94	1.88	0.042	*p* > 0.05
C18:0 iso	0.07	0.07	0	*p* > 0.05
C18:0	12.01	10.24 *	1.251	*p* < 0.05
C18:1 13t	0.08	0.09	0.007	*p* > 0.05
C18:1 9t	2.51	2.13 *	0.268	*p* < 0.05
C18:1 9c	52.18	50.42	1.244	*p* > 0.05
C18:1 10c	2.00	2.98 *	0.692	*p* < 0.05
C18:1 11c	0.38	0.34	0.028	*p* > 0.05
C18:1 12c	0.16	0.21 *	0.035	*p* < 0.05
C20:0	0.03	0.04	0.007	*p* > 0.05
C22:1n9	0.33	0.23 *	0.071	*p* < 0.05
C22:0	0.03	0.02	0.007	*p* > 0.05
9c,13t CLA	0.33	0.21 **	0.085	*p* < 0.01
9c,12t CLA	0.06	0.12 **	0.042	*p* < 0.01
9t,12c CLA	0.05	0.04	0.007	*p* > 0.05
C18:2n6c	2.00	1.88	0.084	*p* > 0.05
9c15c CLA	0.13	0.11	0.014	*p* > 0.05
C18:3 n-6	0.04	0.01 *	0.021	*p* < 0.05
C18:3 n-3	0.10	0.11	0.007	*p* > 0.05
C18:2 n-3	0.10	0.08	0.014	*p* > 0.05
9c 11t CLA	0.19	0.19	0	*p* > 0.05
9t 11t CLA	0.03	0.03	0	*p* > 0.05
C20:2	0.02	0.01	0.007	*p* > 0.05
C20:3n9	0.10	0.11	0.007	*p* > 0.05
UFA, %	60.93	60.65	0.197	*p* > 0.05
SFA, %	38.21	38.40	0.134	*p* > 0.05
PUFA, %	3.15	2.90 **	0.176	*p* < 0.01
MUFA, %	57.68	58.64	0.678	*p* > 0.05
U/S	1.59	1.57	0.014	*p* > 0.05
P/S	0.07	0.07	0	*p* > 0.05
Total mg/g	134.70	222.34 **	61.97	*p* < 0.01

SEM: Stander error of mean; GN: Gannan; TZ: Tianzhu; SFA: Saturated Fatty Acids; UFA: Unsaturated Fatty Acids; MUFA: Monounsaturated Fatty Acids; PUFA: Polyunsaturated Fatty Acids; CLA: Conjugated Linoleic Acid.

**Table 3:** Effects of natural grazing in different regions on rumen fermentation characteristics of Tibetan sheep. Values are presented as mean ± standard error of the mean (SEM) (*n* = 6). Within a row, asterisks denote significant differences between the GN and TZ groups (* *p* < 0.05, ** *p* < 0.01) based on independent samples *t*-test.

Item	Group		SEM	*p*-Value
	TZ	GN		
Total VFA	138.50	147.88 *	6.63	*p* < 0.05
Acetate	66.45	93.29 *	18.97	*p* < 0.05
Propionate	32.97 *	19.02 **	9.86	*p* < 0.01
Isobutyrate	1.03	3.56 *	1.79	*p* < 0.05
Butyrate	36.58	31.48	3.61	*p* > 0.05
Isovalerate	1.02	0.29 **	0.52	*p* < 0.01
Valerate	0.42	0.21	0.14	
Molar proportion, %				
Acetate	47.99	63.09	10.68	*p* > 0.05
Propionate	23.81	12.87	7.74	*p* > 0.05
Isobutyrate	0.74	2.41 **	1.18	*p* < 0.01
Butyrate	26.41	21.29	3.62	*p* > 0.05
Isovalerate	00.74	0.20 **	0.38	*p* < 0.01
Valerate	0.30	0.15	0.11	*p* > 0.05

SEM: Stander error of mean; VFA: Volatile Fatty Acid; GN: Gannan; TZ: Tianzhu.

## Data Availability

The original contributions presented in this study are included in the article/Supplementary Material. Further inquiries can be directed to the corresponding authors.

## Supplementary Materials

The following supporting information can be downloaded at https://www.mdpi.com/article/10.3390/microorganisms14030575/s1: Figure S1: Partial Least Squares Discriminant Analysis (PLS-DA) score plot; Figure S2: Orthogonal Partial Least Squares Discriminant Analysis (OPLS-DA) score plot.
