# Supplementary material for: Meat Quality Differences Between Ganan Tibetan Sheep and Tianzhu Tibetan Sheep Using Metabolomics and Rumen Microbiota Analyses
Source: Microorganisms. 2026 Mar 3;14(3):575. doi: 10.3390/microorganisms14030575 (PMC13028713; doi:10.3390/microorganisms14030575)
Supplement: Supplementary file 1 [file microorganisms-14-00575-s001.zip › microorganisms-4051572-supplementary.pdf]

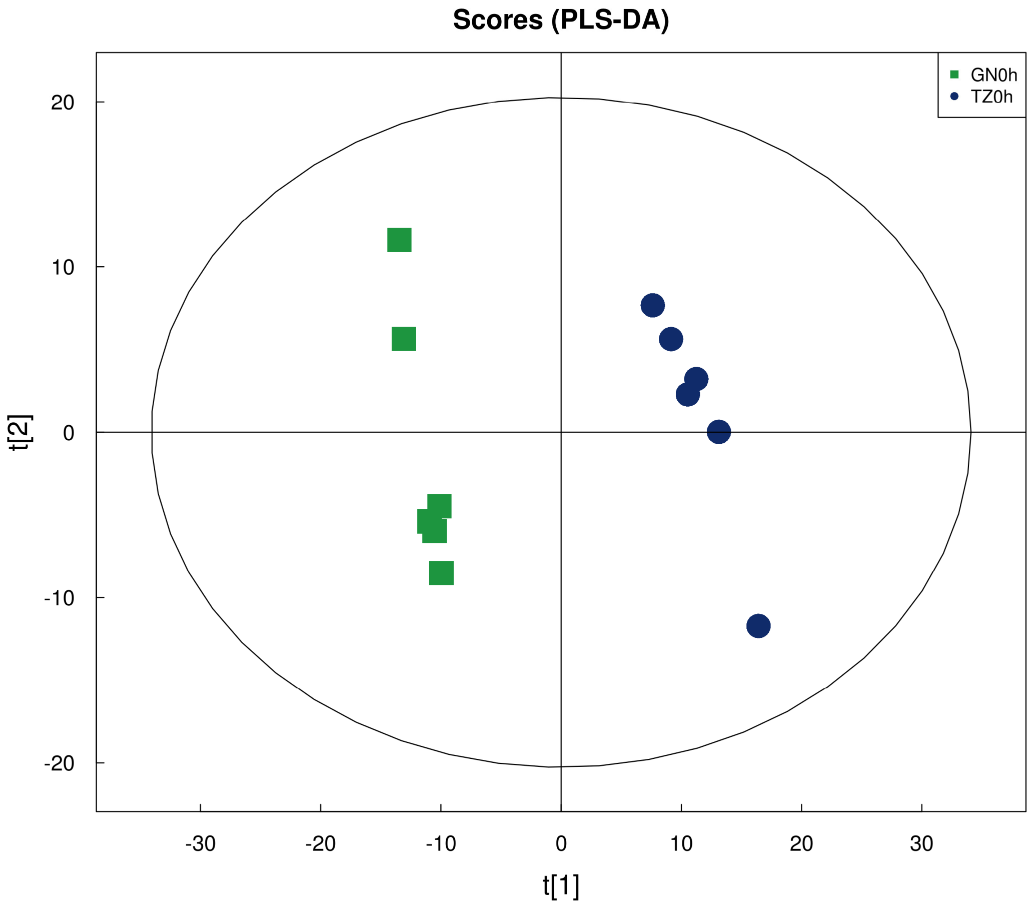

Figure S1. Partial Least Squares Discriminant Analysis (PLS-DA) score plot.

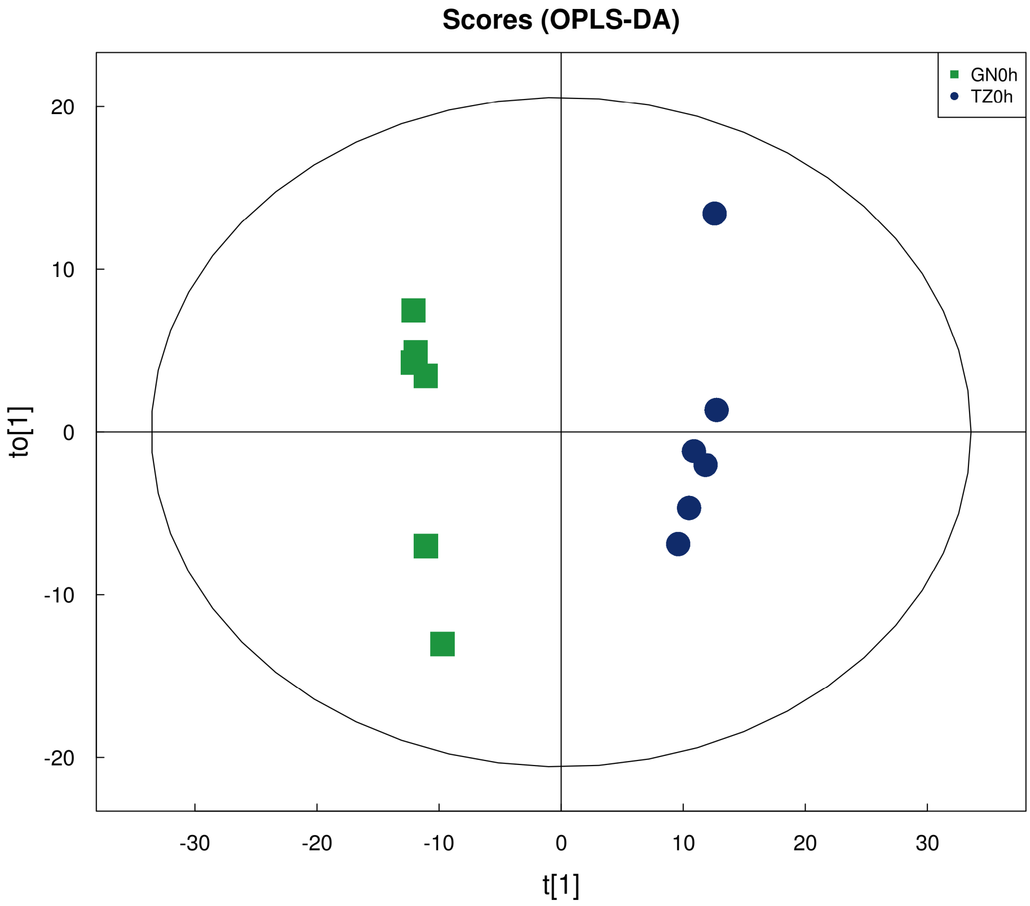

Figure S2. Orthogonal Partial Least Squares Discriminant Analysis (OPLS-DA) score plot.
